# The Institutional Worker

**Published:** 1919-12-06

**Authors:** 


THE
### INSTITUTIONAL WORKER
Being a Special Supplement to "The Hospital"

### OUR BUREAU OF INFORMATION.

### Rules for Correspondents.

1. Every letter must be accompanied by the coupon to be cut from the back cover (inside page) of The Hospital, current issue, and must contain the name and address of the correspondent with pseudonym for publication if desired. Replies by post cannot be given save under exceptional circumstances at the Editor's discretion.

2. Letters from Approved Homes in reply to special needs published in the Bureau should state terms and full particulars, and be sent prepaid under cover to the Editor of the Bureau with name written across coupon for identification.

3. Proprietors of Homes which have not yet been entered on the List of Approved Homes, but have spare accommodation likely to suit special needs, are invited to write for an application form for registration. The fee for registration, which includes two announcements of the Home in the Bureau and other privileges, is 10s.

4. All communications to be addressed to the Editor of The Hospital, 28 Southampton Street, Strand, London, W.C. 2, and marked "Bureau of Information."

### INSTITUTION FACTS AND FIGURES.

### The Question Box.

### REGULATING A PATIENT'S VISITORS.

The question was :

**Elaborate a plan whereby the number of patients' visitors may be regulated on visiting days, so that none may be standing about the corridors and all may see the patient in turn. Each patient to be allowed six visitors, but not more than two at a time.**

**"Rainbow" replies:** When each new patient is admitted to the ward, the sister or staff nurse on duty gives to the nearest relative or friend present two tickets, to be used on visiting days.

The tickets to be something like this :

"John Smith's Hospital,
Watson Ward.

"Admit Bearer to Mrs. Wilson, No. 19.

"*Visiting Days* : Sundays and Wednesdays, 2 to 3; Thursdays (men only), 6 to 7."

Each of these tickets admits one visitor. The ticket is shown to the porter on duty in the entrance hall, and the bearer is then allowed to see the patient. When the first two visitors leave the patient they pass their tickets on to two more visitors, who will be waiting either in the out-patients' waiting-room, or in the grounds attached to the hospital. Failing this, I suppose they must wait in the street.

Should the visiting time be for one hour, the porter will ring the bell each twenty minutes; if for two hours, each forty minutes; and the sister in charge of each ward will see that the visitors present leave the patient unless they know there are no more waiting to visit the patient.

The above method is in use now at a busy hospital with which I am acquainted, and the same acts admirably. With the ticket system there can never be more than two visitors at any one time to the same patient.

**"Matron" replies:**

In order that patients' visitors may be regulated the following plan might be adopted :

On each side of the entrance hall there might be one or two neat rows of pigeon-holes. Each row marked Floor 1 and Floor 2 or Ward or Corridor, as the case may be. Over the rows, in large letters, should be printed "Visitors In" on one side and "Visitors Out" on the other side. Each pigeon-hole should be marked with the number of each bed, and inside each, on the "Visitors In" side, should contain six metal cards, about the size of a lady's visiting-card, numbered 1 to 6 consecutively, and the number of the bed in the corner of each card.

On arrival each visitor is given one card, beginning at No. 1, from the pigeon-hole marked with the number of the bed or room asked for by the visitor, and from the side marked "Visitors In."

On returning the visitor returns the card to the side marked "Visitors Out," and into the the pigeon-hole marked as the card, bed, or room. If the visitor calls and two cards in use have not been returned to "Visitors Out," then the visitor can be asked to wait in the room assigned to visitors. If a visitor arrives and all six cards have been used, that visitor can only go up with special permission. By this system a definite check can be made on all visitors to patients. Complications may arise, such as a visitor forgetting to leave the card, or staying with the patient too long; that can only be avoided by the vigilance of the staff appointed for that particular duty.

**F. Squires replies:**

The following plan for the regulation of a patient's visitors is not put forward as a faultless solution of the problem submitted above. It will, however, be found to work fairly smoothly, providing the conditions stated are possible. Firstly, visitors should have access to the hospital by one entrance only. Visiting hours being usually during the afternoon, an out-patient waiting-room would probably be available as a room for visitors to await their turn. For the working of this scheme it is necessary for the beds to be numbered in each ward : a small enamel label fixed above each bed will serve the purpose.

On the morning of each visiting day the attendant responsible for admitting visitors collects from each ward a list of patients. The lists are headed with the ward number, and are numbered down the left-hand margin "Bed No. 1," and so on. It will be found in practice that one list can be used three or four times before the number of changes necessitates a new one. Metal discs, perforated at the top, and marked "Ward 3, Bed 4" are provided for each bed; they are kept on a hooked board, one row of hooks to each ward. Intending visitors give the patient's name and the attendant issues a disc corresponding to the ward and bed number of the patient as shown on the ward list. Two visitors are admitted with each disc, and no further persons allowed to pass until the disc is returned to the board. It is not possible to limit the total number of visitors to a patient by this plan, and it is left to the patient to arrange for the exchange of his or her visitors. It is advisable for the attendant to commence his duties at least fifteen minutes before the visiting hour, in order that the majority of visitors shall be provided with admission discs by the actual time for visiting. It will be an advantage if the attendant is able to communicate with the wards by telephone without leaving his post.

### Question for December.

### A COTTAGE HOSPITAL LAUNDRY.

For a cottage hospital containing fifty beds, and a staff of twelve including matron, nurses, and maids, give details of size of laundry necessary, also full particulars of the equipment, machinery, and staff required, and some idea as to the cost of working same.

(**A minimum payment of 10s. 6d. will be made for each published answer.**)

RULES.

The following rules must be observed:—

1. Contributions must be written on one side of the paper. Conciseness and terseness are desirable features. The MS. must bear the name and address of the sender and be accompanied by coupon to be cut from the back cover (inside page) of the current issue of THE HOSPITAL. A pseudonym must be chosen if the name is not to be published.

2. Contributions must be addressed to the Editor of THE HOSPITAL Institutional Worker Supplement, 28 & 29 Southampton Street, Strand, London, W.C. 2, should reach him before the end of the current month, and be marked in the left-hand corner "Facts and Figures."

### QUESTIONS INVITED.

IN connection with our Question Box, we offer every month five shillings each for the best questions which are sent in for consideration. The questions may be on any institutional subject and concern any department, from the secretary's to the porter's, or the matron's to the domestic staff; the one essential is that they must be practical and deal with points that have a definite relation to hospital or institutional administration, upkeep, finance, or management. Points relating to artificers' work, housekeeping, the laundry, out-patients, and other practical matters will be welcome. It is hoped that thus, when difficult or doubtful points arise in the course of their work, institutional workers will be encouraged to put them in the form of a question and send them to the Editor, THE HOSPITAL Bureau of Information, 28 & 29 Southampton Street, Strand, London, W.C. 2, marked "Question Box."

The best questions will be published in due course and our readers will be given the opportunity of answering them. Thus all institutional workers may be able to cooperate with the view of helping the work and smoothing the difficulties of each other. In short, we want the Question Box of the *Institutional Worker* to be freely used by every worker habitually.

### ENQUIRIES AND ANSWERS.

### *EMPLOYMENT AND TRAINING.*

### Training-Schools in Bradford.

THE Bradford Royal Infirmary has 220 beds. Candidates are accepted for three years' training after two months' trial. The Bradford Union Hospital has 450 beds. This institution is recognised by the Central Midwives Board as a training-school, and it qualifies candidates who are also certified midwives for the office of superintendent Nurse of the Poor-Law institutions. The training is for a period of three years. Apply to the Matron and Lady Superintendent respectively. —IGOR.

### Nursing of Soldiers' and Sailors' Wives and Families.

WE suggest that you write to the Secretary of the Alexandra Nurses (Soldiers' and Sailors' Families' Association), 23 Queen Anne's Gate, Westminster, S.W., and ask if they have a vacancy. The Association undertakes to supply qualified district nurses for wives and families of soldiers and sailors to any garrison or station (naval or military) at home or abroad where it can be shown that the services of a nurse are required and can be fully utilised.—DEMOBILISED.

### "First Aid" in Glasgow.

THE address of the Ambulance Association in Glasgow is St. Andrew's Ambulance Association, 176 West Regent Street, Glasgow. The objects of the Association are to instruct persons in rendering "first-aid," the provision of ambulances, the organisation of ambulance corps, invalid transport and nursing corps, and generally the promotion of instruction in and carrying out works for the relief of suffering of the sick and injured in peace and war. Write to the General Secretary at the address given.—B. B.

### Demobilised R.A.M.C. Soldier.

THE Army and Navy Male Nurses' Co-operation employs discharged R.A.M.C. men, and we suggest that you apply to the Hon. Secretary at 11A Welbeck Street, London, W. If you prefer, as you suggest, to take a further course of training, apply to the matron of the National Hospital for the Paralysed and Epileptic, Queen Square, Bloomsbury, W.C. This institution provides a two-years' course of training for male nurses.—DEMOBBED.

### Gynæcological Training for Nurse.

YOUR best plan will be to write for advice upon this matter to the matrons of some well-known women's hospitals, as, for instance, the South London Hospital for Women, Clapham Common, S.W., The Chelsea Hospital for Women, Chelsea, S.W., and the Samaritan Hospital for Women, Marylebone Road, N.W.—TRAINED NURSE.

### *MISCELLANEOUS.*

### Address Wanted.

THE address of the National Food Reform Association, or, rather, the Food Education Society, with which the Association is now amalgamated, is Dane's Inn House, 265 Strand, W.C. 2.—K. A. T.

### Hospital Water-Taps.

WE have referred to this difficulty in these columns before. Where stop cocks are not available at suitable points the whole water service of the building must necessarily be interfered with in replacing a washer. The only remedies are either to fix stopcocks at suitable points in each section of the supply or to replace taps with that type which can be repaired without shutting off the water. The "Tapval" tap, manufactured by Messrs. Tonkin, Collins & Co., Ltd., of 116 Victoria Street, Westminster, S.W., is the ideal tap for this purpose. It is so constructed that in taking out the jumper to renew the washer the water is instantly and automatically cut off in the tap.—T. S. H.

### A Guild of Service.

WE think that the Guild of Service, a Service which is intended to help the spiritual life of the officers and attendants in Local Government Board institutions, would interest you. The Guild is anxious to obtain the names of those who would be willing to become associates or act as local secretaries. If this suggestion appeals to you, write to the Hon. Secretary, Miss M. A. King, 34 West Hill, Sydenham, S.E. 26.—"VERA."

### Pay Patients in Free Wards.

WE think that, in practice, you would not find it convenient or desirable to place in your free wards patients paying a fixed contribution for their maintenance. It must be admitted that many patients who come to the free wards of a hospital can contribute towards their maintenance, but at the same time have not sufficient means to pay even the minimum cost for treatment in a private institution. The question is one, of course, which depends upon the constitution of your hospital, and if that does not provide the treatment of paying patients, it would be essential for you to get the objects of your institution amended. This, doubtless, could be done at your general meeting or at such meeting as the rules provide; but before taking this step you ought to consult the members of your staff, and possibly all the medical practitioners residing in your neighbourhood. On the whole, the best course would seem to be to inquire into the circumstances of the patients and to urge upon all those who can afford to do so their duty to contribute in accordance with their means towards the cost of maintenance.—W. A. R. E.

